# Validation of Hyponatremia as a Prognostic Predictor in Multiregional Upper Tract Urothelial Carcinoma

**DOI:** 10.3390/jcm9041218

**Published:** 2020-04-23

**Authors:** Hsin-Chih Yeh, Ching-Chia Li, Sheng-Chen Wen, Nirmish Singla, Solomon L. Woldu, Haley Robyak, Chun-Nung Huang, Hung-Lung Ke, Wei-Ming Li, Hsiang-Ying Lee, Chia-Yang Li, Bi-Wen Yeh, Sheau-Fang Yang, Hung-Pin Tu, Shahrokh F. Shariat, Arthur I. Sagalowsky, Jay D. Raman, Yair Lotan, Jer-Tsong Hsieh, Vitaly Margulis, Wen-Jeng Wu

**Affiliations:** 1 Department of Urology, Kaohsiung Municipal Ta-Tung Hospital, Kaohsiung 80145, Taiwan; patrick1201.tw@yahoo.com.tw (H.-C.Y.); cnhuang.uro@gmail.com (C.-N.H.); ashum1009@hotmail.com (H.-Y.L.); 2 Department of Urology, Kaohsiung Medical University Hospital, Kaohsiung 80756, Taiwan; ccli1010@hotmail.com (C.-C.L.); carl0815@gmail.com (S.-C.W.); hunglungke@yahoo.com.tw (H.-L.K.); u8401067@yahoo.com.tw (W.-M.L.); bewen90@yahoo.com.tw (B.-W.Y.); 3 Department of Urology, School of Medicine, College of Medicine, Kaohsiung Medical University, Kaohsiung 80708, Taiwan; 4 Department of Urology, University of Texas Southwestern Medical Center, Dallas, TX 75390, USA; nirmish@gmail.com (N.S.); solomon.woldu@utsouthwestern.edu (S.L.W.); sfshariat@gmail.com (S.F.S.); arthur.sagalowsky@utsouthwestern.edu (A.I.S.); yair.lotan@utsouthwestern.edu (Y.L.); 5 Division of Urology, Department of Surgery, Penn State Health Milton S. Hershey Medical Center, Hershey, PA 17033, USA; hrobyak@gmail.com (H.R.); jraman@pennstatehealth.psu.edu (J.D.R.); 6 Graduate Institute of Medicine, College of Medicine, Kaohsiung Medical University, Kaohsiung 80708, Taiwan; chiayangli@kmu.edu.tw (C.-Y.L.); sfyang@kmu.edu.tw (S.-F.Y.); 7 Department of Pathology, Kaohsiung Medical University Hospital, Kaohsiung Medical University, Kaohsiung 80708, Taiwan; 8 Department of Public Health and Environmental Medicine, School of Medicine, College of Medicine, Kaohsiung Medical University, Kaohsiung 80708, Taiwan; p915013@kmu.edu.tw; 9 Department of Urology, Medical University of Vienna 1090, Vienna, Austria

**Keywords:** upper tract urothelial carcinoma, hyponatremia, ECOG, progression, prognosis

## Abstract

Hyponatremia has been shown to be associated with prognosis in various cancers, but its role in upper tract urothelial carcinoma (UTUC) is largely unidentified. We created an international multiregional cohort of UTUC, consisting of 524 and 213 patients from Taiwan and the U.S., to validate the significance of hyponatremia. Clinicopathologic characteristics were compared according to the presence of hyponatremia. Univariate and multivariate Cox regression models were used to investigate the association of hyponatremia with disease progression and survival. The impact of hyponatremia in patients from distinct regions was also analyzed. Hyponatremia was found in 143 (19.4%) patients. Hyponatremic patients had significantly worse Eastern Cooperative Oncology Group (ECOG) performance status (*p* = 0.00001) and higher pT stage (*p* = 0.002). In multivariate analysis, hyponatremia was an independent prognostic factor for progression (HR 1.585, 95% CI 1.115–2.253, *p* = 0.010), cancer-specific death (HR 2.225, 95% CI 1.457–3.397, *p* = 0.0002), and overall mortality (HR 1.819, 95% CI 1.299–2.545, *p* = 0.0005). Kaplan–Meier analysis showed the consistent adverse effect of hyponatremia on all outcomes in patients from Taiwan and the U.S. (all *p* < 0.05). Hyponatremia is commonly accessible and can serve as a negative marker for both the general health condition and disease severity of UTUC patients. A similar implication of hyponatremia in progression and survival despite patients’ region of presentation suggests its general applicability across different ethnicities.

## 1. Introduction

Upper tract urothelial carcinoma (UTUC) arises from the epithelial lining of pelvicalyceal cavities and ureter. UTUC is highly heterogeneous and generally accounts for 5–10% of all urothelial carcinomas but can be as high as 30% in Taiwan. In addition to an epidemiologic difference, it was reported that disease features and predictors of oncologic outcomes varied according to patients’ geographic distribution [1,2]. The divergence may lead to the inaccurate prognostication of formerly established predictive models when extrapolating to different populations. Therefore, it is important to incorporate patients of diverse ethnicities from distinct regions when investigating the potential generalizability of each factor.

An imprecision of clinical staging for UTUC is not uncommon despite advances in imaging diagnostic techniques [3]. Furthermore, a wide range of outcomes among patients in an identical prognostic group is observed in the clinical practice. Hence, traditional prognosticators are inadequate to achieve accurate risk assessment [4]. Additionally, many pathologic factors are confirmed only after extirpative surgery, limiting their utility as perioperative predictors. Preoperative prognostication can help determine if a patient may benefit from more aggressive treatment, such as neoadjuvant therapy, extended lymph node dissection, or multidisciplinary management.

While several preoperative factors are not routinely measured, serum sodium concentration is almost universally tested as part of laboratory assessment before surgery. In an analysis of 3357 hospitalized cancer patients, Doshi et al. found that those with hyponatremia had longer hospitalization and higher mortality [5]. Specifically, low sodium level was identified as a negative prognostic factor in various malignancies, including renal cell carcinoma [6], small cell lung cancer [7], non-small cell lung cancer [8,9], breast cancer [9], gastric cancer [10], hepatocellular carcinoma [11], colorectal cancer [9,12], lymphoma [9,13], and malignant pleural mesothelioma [14].

In Japan, Fujita et al. found that serum sodium <141 mmol/L was predictive of cancer-specific mortality in 139 patients with UTUC but failed to predict disease recurrence [15]. The author further used preoperative sodium and hemoglobin to perform risk classification to show survival differences in 226 Japanese patients with UTUC [16]. However, more than 90% cases of the low serum sodium group in their pilot study were not hyponatremic [15], which failed to reflect actual clinical relevance. Moreover, the significance of definite hyponatremia has not been examined or validated in patients from other regions. Therefore, the purpose of the present study was to evaluate the impact of hyponatremia on the progression and survival of UTUC. We also analyzed the respective influence of hyponatremia in patients from different regions.

## 2. Materials and Methods

### 2.1. Patient Collection

Between 1997 and 2017, 524 and 213 patients surgically treated with curative intent for UTUC at Kaohsiung Medical University Healthcare System in Taiwan and at 2 tertiary care medical facilities in the U.S., respectively, were enrolled. This study was approved by the review board of our institution (KMUHIRB-E(I)-20180214) and other two participating hospitals. We retrospectively collected information on the demographic characteristics, pathologic features and oncologic follow up. Patients who had evidence of distant metastasis at diagnosis or incomplete clinicopathologic data were excluded. Parameters including age, gender, Eastern Cooperative Oncology Group (ECOG), estimated glomerular filtration rate, history of bladder cancer, tumor location, hydronephrosis, type and approach of extirpative surgery, tumor focality, grade, pT stage, lymphovascular invasion, lymph node involvement, and hyponatremia were recorded. The type and approach of extirpative surgery were based on surgeon discretion by considering tumor location, size, likelihood of progression, and surgeon experience. Systemic chemotherapy was administered after taking patients’ tumor stage, performance status, renal function, and willingness into account. Laboratory data were measured within 2 weeks preoperatively, and hyponatremia was defined as <136 mmol/L by the U.S. guideline [17].

### 2.2. Postoperative Follow-Up

Patients were regularly followed postoperatively. Investigations included physical examination, urinalysis, urine cytology, cystoscopy, blood tests, and periodic imaging studies. Follow-up assessment was performed every 3 months in the first year. Patients from the U.S. were followed every 6 months in the second year and every 12 months since the third year after surgery. Patients from Taiwan were assessed semiannually and annually since the third and the fifth year. Tumors relapsing in the operation site or distant organ but not in the bladder or contralateral upper tract were considered as disease progression. The cause of mortality was determined by the treating physician, medical chart review, or death certificate.

### 2.3. Statistical Analysis

Statistical analysis was performed using SPSS^®^ version 22.0. Patients were grouped according to the presence of hyponatremia. Independent Student’s *t* test and chi-square test were used to evaluate the association of hyponatremia with continuous and categorical covariates, respectively. The end points were progression-free survival (PFS), cancer-specific survival (CSS), and overall survival (OS) calculated from the date of extirpative surgery to the date at which an event or censoring occurred. Survival curves were plotted by the Kaplan–Meier method, and prognostic differences were compared using the log rank test. The implication of hyponatremia in outcomes was also analyzed based on the country of presentation to test its generalizable potential. Univariate and multivariate Cox regression models were performed to identify predictive factors for PFS, CSS, and OS, and two-tailed *p* < 0.05 was considered statistically significant for all analyses.

## 3. Results

### 3.1. Clinicopathologic Characteristics

The clinicopathologic characteristics of 737 patients are listed in Table 1. The mean age and follow up was 68.0 years and 37.9 months. Radical nephroureterectomy was performed in 630 (85.5%) cases, and 389 (52.8%) of this cohort were operated with an open method. Three hundred and eight (41.8%) tumors were located in the renal pelvis, 309 (41.9%) were located in the ureter, and 120 (16.3%) were in both locations. Histological grade was classified as high in 608 (82.5%) tumors, and stage distribution was pTa/Tis, pT1, pT2, pT3, and pT4 in 165 (22.4%), 150 (20.4%), 154 (20.9%), 225 (30.5%), and 43 (5.8%), respectively. Lymphovascular invasion and lymph node metastasis were observed in 178 (24.2%) and 67 (9.1%) patients. Disease progression, cancer-specific death, and all-cause mortality occurred in 187 (25.4%), 130 (17.6%), and 200 (27.1%) cases. In all, 143 (19.4%) patients had hyponatremia preoperatively. Hyponatremia was significantly associated with age (*p* = 0.006), region of presentation (*p* = 0.00007), ECOG (*p* = 0.00001), estimated glomerular filtration rate (*p* = 0.043), type of surgery (*p* = 0.010), and pT stage (*p* = 0.002). There were no statistical differences in terms of gender, history of bladder cancer, tumor location, hydronephrosis, surgery approach, focality, grade, lymphovascular invasion, and lymph node status between the two groups. Patients’ characteristics according to regions (Taiwan or U.S.) are shown in Table S1.

### 3.2. Correlation of Hyponatremia with Outcomes of UTUC Patients

Table 2 showed the results of Cox regression analyses. In univariate analysis, significant predictors for lower PFS, CSS, and OS were older age (*p* = 0.026, 0.001, and 0.00005, respectively), worse ECOG (*p* = 0.029, *p* = 0.035, and *p* < 0.00001, respectively), concurrent pelvicalyceal and ureteral tumors (*p* = 0.001, *p* = 0.00004, and *p* < 0.00001, respectively), hydronephrosis (*p* = 0.015, 0.035, and 0.033, respectively), open surgery (*p* = 0.023, 0.004, and 0.008, respectively), multifocality (*p* = 0.0004, 0.0001, and 0.00003, respectively), grade (*p* < 0.00001, *p* = 0.00003, and *p* < 0.00001, respectively), pT stage (all *p* < 0.00001), lymphovascular invasion (all *p* < 0.00001), lymph node involvement (*p* < 0.00001, *p* = 0.00001, and *p* < 0.00001, respectively), and hyponatremia (*p* = 0.001, 0.0002, and 0.00001, respectively). In multivariate analysis, age was significantly associated with CSS (*p* = 0.036) and OS (*p* = 0.008) but not with PFS, and lymphovascular invasion was significantly predictive of PFS (*p* = 0.012) and CSS (*p* = 0.007) but not of OS. Grade was significant only for PFS (*p* = 0.023), and the significance of ECOG was merely for OS (*p* = 0.002). Consistent independent prognosticators for PFS, CSS, and OS in the current cohort were pT stage (all *p* < 0.00001), lymph node status (*p* = 0.003, 0.048, and 0.028, respectively), and hyponatremia (*p* = 0.010, 0.0002, and 0.0005, respectively). The results of analysis using sodium level as a continuous variable were shown in Table S2.

In addition, we did not include adjuvant chemotherapy in the multivariate adjustment, because this variable was not provided by one of the participating hospitals. We analyzed the remaining 648 patients who had records of adjuvant chemotherapy, and an independent prognostic effect of hyponatremia on PFS, CSS, and OS remained after adding adjuvant chemotherapy as a covariate in the multivariate analysis (HR 1.472, 95% CI 1.022–2.121, *p* = 0.038; HR 2.190, 95% CI 1.412–3.396, *p* = 0.0005; HR 1.773, 95% CI 1.247–2.520, *p* = 0.001; respectively).

### 3.3. Effect of Hyponatremia on Outcomes in Different Ethnicities

**Figure 1:**
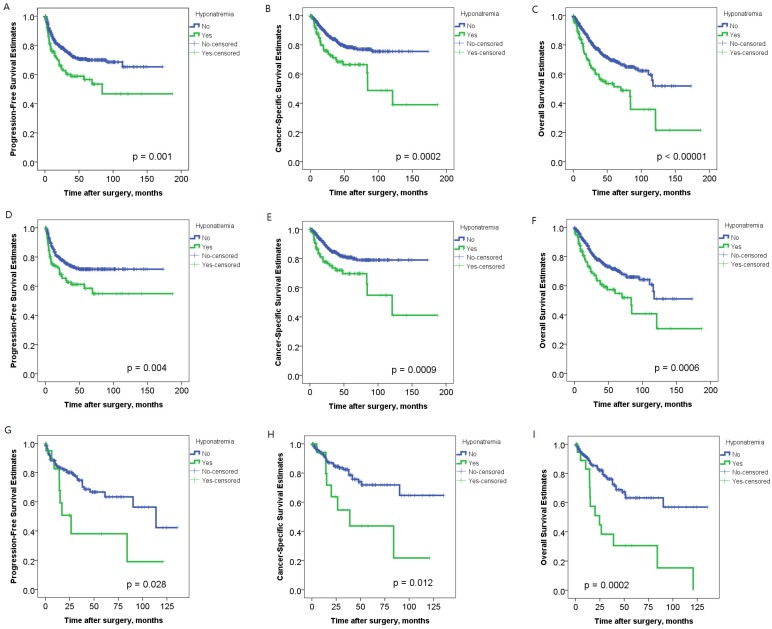
In Kaplan–Meier analysis, hyponatremia was significantly associated with lower progression-free survival (PFS), cancer-specific survival (CSS) and overall survival (OS) in the whole cohort of 737 patients with UTUC (**A**–**C**), and in patients with UTUC either from Taiwan (**D**–**F**) or the U.S. (**G**–**I**).

The five-year PFS, CSS, and OS rates according to preoperative serum sodium level were 70.5%, 78.4%, and 69.5% for patients without hyponatremia, and 56.5%, 66.5%, and 51.3% for hyponatremic patients, respectively. Kaplan–Meier curves of PFS, CSS, and OS were compared and showed a clear difference between the two groups (Figure 1A–C, *p* = 0.0007, *p* = 0.0002, and *p* <0.00001, respectively). The prognostic significance of hyponatremia was also analyzed based on patients’ geographic distribution. Hyponatremia was associated with decline in PFS, CSS, and OS in patients from Taiwan (Figure 1D–F, *p* = 0.004, 0.0009, and 0.0006, respectively) and the U.S. (Figure 1G–I, *p* = 0.028, 0.012, and 0.0002, respectively), implying its general applicability by the consistent impact on UTUC, regardless of country of presentation.

## 4. Discussion

Hyponatremia is not infrequent in cancer patients and is a clinically useful biomarker due to its easy accessibility. Since hospital-acquired hyponatremia is common for cancer patients, we did not use the lowest sodium level during patients’ hospital stay but the serum sodium measured preoperatively before intravenous infusion of fluids to define hyponatremia. UTUC is a rare malignancy worldwide, and the role of hyponatremia in UTUC is largely undetermined. Although hyponatremia was proposed as a prognosticator for Japanese UTUC, no previous studies have investigated the implication of hyponatremia in UTUC from different regions. It is crucial to identify significant predictors available preoperatively to enhance the risk stratification and decision making. From this multiregional cohort of UTUC, we found that hyponatremia was an independent prognostic factor for disease progression and survival. Moreover, the significance of hyponatremia in all outcomes was found both in Taiwanese and American populations.

Little is known about hyponatremia in cancer patients. The frequency of hyponatremia and its impact on cancer may diverge owing to variations in definition. As mentioned, we did not consider patients who developed hyponatremia only after admission to preclude any fluid-related effect. Discrepancies may also occur with an inconsistent cutoff value to describe low sodium concentration in cancer patients. For example, 138 and 139 mmol/L have been used as the threshold in renal cell carcinoma [6,18], and 133 mmol/L was employed in gastric cancer [10]. A continuous form of sodium level was included in the multivariate analysis of UTUC patients from Japan, and natremia was significantly associated with CSS [15]. A value below the median of their cohort, 141 mmol/L, was used to define low natremia in the Cox regression and risk classification model [15,16]. However, this dichotomization could possibly be flawed by the lack of clinical pertinence, since it is of doubt whether a sodium level in the lower half of the normal physiological range is detrimental or relevant to cancer progression. Thus, in this study, <136 mmol/L, a common standard in the U.S. guideline, was used as the definition of hyponatremia [17].

Hyponatremia can be caused by medical comorbidities, including heart failure, liver cirrhosis, and renal failure, and therefore lead to higher mortality. However, the worsening of underlying disease is perhaps not the only reason why hyponatremia links to diminished survival, because a persistent inverse relationship between sodium concentration and mortality after adjusting for comorbid conditions has been repeatedly reported [6,12,19,20,21,22]. Hyponatremia is also postulated to result in a worse outcome through disturbing 3-dimensional conformations of proteins, metabolic equilibrium, and genetic balance [5,21]. Consequently, it remains elusive that hyponatremia-associated mortality is related to the nature of underlying illness or hyponatremia itself.

Hyponatremia may reflect the general condition of UTUC patients, as evidenced by its significant association with ECOG performance status. ECOG was an assessment for cancer patients’ well-being as well as functionality, and it was closely connected to their quality of life and comorbidities. A negative correlation of serum sodium level with ECOG has also been described in lung cancer [23], and improved performance status by hyponatremia correction may lead to better acceptance for anti-cancer therapy with resultant greater OS [24]. On the other hand, hyponatremia could possibly indicate biologically aggressive UTUC, because it was significantly associated with adverse pT stage and tumor-specific outcomes, such as PFS and CSS. Similarly, metastatic small cell lung cancer patients had a higher risk of developing hyponatremia [7], and sodium could be restored to near-normal levels after effective treatment [25]. The independently predictive value of hyponatremia after multivariate adjustment for all outcomes of UTUC may support it to be a surrogate indicator of patients’ general health and cancer severity. In comparison, our results showed that ECOG well predicted OS but not tumor-related features such as pT stage, PFS, and CSS for patients with UTUC, which has also been reported in a previous publication [26].

Hyponatremia may not only restrict the use of chemotherapy due to massive hydration but also adversely affect treatment response and the survival of cancer patients. In non-Hodgkin’s lymphoma, patients with hyponatremia had shorter remission duration and poorer outcome than those who were eunatremic [13]. Likewise, hyponatremia was a significant predictor of worse prognosis in metastatic renal cell carcinoma patients receiving immunotherapy or molecular targeted therapy [18,22,27]. Compared to patients without hyponatremia, our results showed that hyponatremic UTUC patients had a similar trend for worse outcomes in cases receiving adjuvant chemotherapy (*p* = 0.097 and 0.036 for CSS and OS, respectively). Interestingly, a study mentioned that the benefit of adjuvant chemotherapy in UTUC is significant in Japanese patients who had low sodium or hemoglobin concentration but not in those had normal levels [28]. However, the effect of adjuvant chemotherapy was similar in patients with and without hyponatremia in this cohort.

Although the syndrome of inappropriate antidiuretic hormone secretion, driven by ectopic tumor-producing arginine vasopressin, is the most common cause of hyponatremia directly related to malignancies, it affects only 1%–2% of the entire cancer population [29]. Except for small cell lung cancer, head and neck cancer, and lymphoma, inappropriate antidiuretic hormone secretion is rare in cancers and simply described in case reports [30]. As for hyponatremia of other conditions, the optimal treatment for hyponatremia of cancer patients is to correct the underlying cause of each classified category, which is based on the tonicity and volume status [17,30]. Importantly, Waikar et al. found a consistently significant increase in the risk of death from a range of hyponatremia in patients with malignancies but not in those with sepsis, pneumonia, liver, or respiratory diseases [21]. Furthermore, the resolution of hyponatremia has been associated with better survival in cancer patients [5,7,12,21,24]. Taken together, cancer patients are reasonable targets to investigate the fundamental pathophysiology of hyponatremia and the potential benefit from normalizing sodium concentration in future prospective studies.

The study is limited by its retrospective nature and lack of a uniform treatment protocol. Heterogeneity existed in this multiregional population, and we attempted to correct as many confounding factors as possible. However, we were unable to collect detailed medication history or thorough underlying comorbidities of the patients. Only patients’ renal function and performance status were adjusted in the analysis. Postoperative serum sodium was not routinely measured, and thus a potential prognostic implication of sodium alteration and the possible benefit of hyponatremia resolution in UTUC patients were unknown. Nevertheless, the significance and general applicability of preoperative hyponatremia could be strengthened by the sizable cohort and external validation in different populations.

## 5. Conclusions

Serum sodium level is readily available for most patients before major surgeries. We demonstrate that hyponatremia independently predicts disease progression and the survival of patients with UTUC. Preoperative hyponatremia is also suggestive of a universally applicable marker, since its significance is validated in patients from distinct regions. Including hyponatremia in the risk stratification model is promising to refine our treatment strategy for patients with UTUC.

## Figures and Tables

**Table 1:** Association of hyponatremia with patients’ clinicopathologic factors.

Variable	Category/Value	No. of Patients (%)	Hyponatremia		*p* Value
			No	Yes	
Age, years (mean, SD)	68.0 (10.5)	737 (100.0)	67.5 (10.1)	70.2 (11.7)	0.006
Gender	Female	361 (49.0)	295 (49.7)	66 (46.2)	0.451
	Male	376 (51.0)	299 (50.3)	77 (53.8)	
Region	Taiwan	524 (71.1)	403 (67.8)	121 (84.6)	0.00007
	U.S.	213 (28.9)	191 (32.2)	22 (15.4)	
ECOG	≤1	603 (81.8)	504 (84.8)	99 (69.2)	0.00001
	≥2	134 (18.2)	90 (15.2)	44 (30.8)	
eGFR, mL/min/1.73 m^2^ (mean, SD)	51.8 (27.8)	737 (100.0)	52.8 (27.9)	47.6 (26.7)	0.043
History of bladder cancer	No	527 (71.5)	418 (70.4)	109 (76.2)	0.164
	Yes	210 (28.5)	176 (29.6)	34 (23.8)	
Tumor location	Renal pelvis	308 (41.8)	247 (41.6)	61 (42.7)	0.528
	Ureter	309 (41.9)	254 (42.8)	55 (38.5)	
	Both	120 (16.3)	93 (15.7)	27 (18.9)	
Hydronephrosis	No	307 (41.7)	243 (40.9)	64 (44.8)	0.402
	Yes	430 (58.3)	351 (59.1)	79 (55.2)	
Type of surgery	Nephroureterectomy	630 (85.5)	498 (83.8)	132 (92.3)	0.010
	Distal ureterectomy	107 (14.5)	96 (16.2)	11 (7.7)	
Approach of surgery	Open	389 (52.8)	311 (52.4)	78 (54.5)	0.638
	Laparoscopy	348 (47.2)	283 (47.6)	65 (45.5)	
Focality	Unifocal	521 (70.7)	420 (70.7)	101 (70.6)	0.985
	Multifocal	216 (29.3)	174 (29.3)	42 (29.4)	
Grade	Low	129 (17.5)	110 (18.5)	19 (13.3)	0.139
	High	608 (82.5)	484 (81.5)	124 (86.7)	
pT stage	pTa/Tis	165 (22.4)	147 (24.7)	18 (12.6)	0.002
	pT1	150 (20.4)	123 (20.7)	27 (18.9)	
	pT2	154 (20.9)	111 (18.7)	43 (30.1)	
	pT3	225 (30.5)	182 (30.6)	43 (30.1)	
	pT4	43 (5.8)	31 (5.2)	12 (8.4)	
Lymphovascular invasion	No	559 (75.8)	448 (75.4)	111 (77.6)	0.581
	Yes	178 (24.2)	146 (24.6)	32 (22.4)	
pN stage	pN0	277 (37.6)	219 (36.9)	58 (40.6)	0.488
	pNx	393 (53.3)	323 (54.4)	70 (49.0)	
	pN+	67 (9.1)	52 (8.8)	15 (10.5)	
Progression	No	550 (74.6)	458 (77.1)	92 (64.3)	0.002
	Yes	187 (25.4)	136 (22.9)	51 (35.7)	
Death of UTUC	No	607 (82.4)	503 (84.7)	104 (72.7)	0.001
	Yes	130 (17.6)	91 (15.3)	39 (27.3)	
All-cause death	No	537 (72.9)	453 (76.3)	84 (58.7)	0.00002
	Yes	200 (27.1)	141 (23.7)	59 (41.3)	

SD: standard deviation, ECOG: Eastern Cooperative Oncology Group, eGFR: estimated glomerular filtration rate, UTUC: upper tract urothelial carcinoma.

**Table 2:** Univariate and multivariate analyses of progression-free, cancer-specific, and overall survival in 737 patients with UTUC.

Variable	Progression-Free Survival						Cancer-Specific Survival						Overall Survival					
	Univariate Analysis			Multivariate Analysis			Univariate Analysis			Multivariate Analysis			Univariate Analysis			Multivariate Analysis		
	HR	95% CI	*p* Value	HR	95% CI	*p* Value	HR	95% CI	*p* Value	HR	95% CI	*p* Value	HR	95% CI	*p* Value	HR	95% CI	*p* Value
**Age (continuous)**	1.016	1.002–1.031	0.026	1.008	0.993–1.023	0.281	1.029	1.011–1.048	0.001	1.020	1.001–1.039	0.036	1.030	1.016–1.045	0.00005	1.020	1.005–1.034	0.008
**Gender**																		
Female	1		0.096	1		0.762	1		0.086	1		0.871	1		0.051	1		0.619
Male	1.277	0.957–1.704		1.048	0.775–1.417		1.355	0.958–1.916		1.031	0.715–1.485		1.320	0.999–1.745		1.077	0.804–1.441	
**Region**																		
Taiwan	1		0.385	1		0.269	1		0.080	1		0.044	1		0.222	1		0.169
U.S.	1.154	0.836–1.593		1.265	0.834–1.918		1.395	0.960–2.026		1.651	1.014–2.687		1.214	0.889–1.658		1.332	0.885–2.006	
**ECOG**																		
≤1	1		0.029	1		0.082	1		0.035	1		0.596	1		<0.00001	1		0.002
≥2	1.464	1.041–2.059		1.384	0.960–1.996		1.545	1.031–2.316		1.128	0.722–1.763		2.129	1.568–2.890		1.702	1.221–2.371	
**eGFR (continuous)**	0.998	0.993–1.004	0.534	1.000	0.994–1.006	0.949	0.997	0.991–1.003	0.353	1.000	0.992–1.008	0.980	0.997	0.992–1.002	0.269	1.000	0.994–1.006	0.979
**History of bladder cancer**																		
No	1		0.247	1		0.130	1		0.195	1		0.122	1		0.270	1		0.216
Yes	1.201	0.881–1.639		1.291	0.927–1.799		1.276	0.882–1.844		1.366	0.920–2.029		1.186	0.875–1.607		1.227	0.887–1.699	
**Tumor location**																		
Renal pelvis	1		0.001	1		0.162	1		0.00004	1		0.211	1		<0.00001	1		0.288
Ureter	1.106	0.796–1.538		1.195	0.815–1.753		1.069	0.710–1.610		1.044	0.650–1.679		1.193	0.861–1.653		1.141	0.783–1.662	
Both	1.970	1.351–2.872		1.639	0.986–2.724		2.469	1.600–3.812		1.650	0.904–3.011		2.352	1.641–3.371		1.487	0.908–2.434	
**Hydronephrosis**																		
No	1		0.015	1		0.718	1		0.035	1		0.071	1		0.033	1		0.129
Yes	1.457	1.076–1.973		1.066	0.753–1.509		1.463	1.027–2.084		1.504	0.966–2.341		1.361	1.025–1.806		1.307	0.925–1.847	
**Type of surgery**																		
Nephroureterectomy	1		0.110	1		0.528	1		0.248	1		0.830	1		0.132	1		0.609
Distal ureterectomy	0.666	0.404–1.097		0.815	0.432–1.539		0.705	0.389–1.277		0.919	0.424–1.990		0.689	0.424–1.118		0.851	0.458–1.581	
**Approach of surgery**																		
Open	1		0.023	1		0.534	1		0.004	1		0.762	1		0.008	1		0.460
Laparoscopy	0.712	0.531–0.955		1.119	0.785–1.595		0.586	0.408–0.841		1.072	0.683–1.685		0.679	0.510–0.904		1.140	0.806–1.612	
**Focality**																		
Unifocal	1		0.0004	1		0.843	1		0.0001	1		0.981	1		0.00003	1		0.645
Multifocal	1.712	1.273–2.303		1.041	0.698–1.552		1.994	1.407–2.827		0.994	0.607–1.628		1.830	1.378–2.430		1.099	0.736–1.640	
**Grade**																		
Low	1		<0.00001	1		0.023	1		0.00003	1		0.170	1		<0.00001	1		0.106
High	5.517	2.822–10.786		2.268	1.118–4.602		5.844	2.573–13.274		1.847	0.770–4.432		3.186	1.934–5.247		1.564	0.909–2.692	
**pT stage**																		
pTa/Tis	1		<0.00001	1		<0.00001	1		<0.00001	1		<0.00001	1		<0.00001	1		<0.00001
pT1	1.730	0.823–3.637		1.665	0.775–3.577		0.938	0.329–2.675		0.895	0.306–2.617		0.921	0.495–1.713		0.933	0.491–1.774	
pT2	3.179	1.614–6.260		2.442	1.189–5.016		2.455	1.025–5.879		1.832	0.725–4.629		1.705	0.987–2.946		1.375	0.759–2.492	
pT3	7.450	3.988–13.916		4.634	2.365–9.080		8.600	3.959–18.681		5.146	2.221–11.928		3.961	2.446–6.413		2.786	1.621–4.786	
pT4	18.286	9.086–36.802		6.111	2.668–13.998		26.655	11.503–61.764		7.482	2.735–20.465		10.728	6.025–19.102		3.809	1.830–7.928	
**Lymphovascular invasion**																		
No	1		<0.00001	1		0.012	1		<0.00001	1		0.007	1		<0.00001	1		0.058
Yes	3.253	2.437–4.343		1.544	1.101–2.163		4.055	2.867–5.735		1.729	1.158–2.581		2.646	1.987–3.524		1.384	0.989–1.938	
**pN stage**																		
pN0	1		<0.00001	1		0.003	1		0.00001	1		0.048	1		<0.00001	1		0.028
pNx	0.805	0.581–1.116		0.941	0.672–1.317		0.804	0.539–1.198		0.953	0.631–1.441		0.790	0.580–1.076		0.944	0.685–1.301	
pN+	4.287	2.892–6.354		2.021	1.259–3.244		4.818	3.038–7.641		1.822	1.036–3.204		3.441	2.307–5.134		1.751	1.080–2.839	
**Hyponatremia**																		
No	1		0.001	1		0.010	1		0.0002	1		0.0002	1		0.00001	1		0.0005
Yes	1.735	1.257–2.394		1.585	1.115–2.253		2.030	1.394–2.955		2.225	1.457–3.397		1.974	1.456–2.677		1.819	1.299–2.545	

HR: hazard ratio, CI: confidence interval, ECOG: Eastern Cooperative Oncology Group, eGFR: estimated glomerular filtration rate.

## Supplementary Materials

The following are available online at https://www.mdpi.com/2077-0383/9/4/1218/s1, Table S1: Patients’ characteristics according to regions of presentation, Table S2: Univariate and multivariate analyses of progression-free, cancer-specific and overall survival in 737 patients with UTUC (using sodium level as a continuous variable).
