# Supplementary material for: Validation of Hyponatremia as a Prognostic Predictor in Multiregional Upper Tract Urothelial Carcinoma
Source: J Clin Med. 2020 Apr 23;9(4):1218. doi: 10.3390/jcm9041218 (PMC7231247; doi:10.3390/jcm9041218)
Supplement: Supplementary file 1 [file jcm-09-01218-s001.pdf]

Table S1. Patients' characteristics according to regions of presentation.

Variable	Category / Value	No. of Patients (%)	Region		*p* value
			Taiwan	U.S.	
Age, years (mean, SD)	68.0 (10.5)	737 (100.0)	67.8 (10.6)	68.5 (10.2)	0.451
Gender	Female	361 (49.0)	291 (55.5)	70 (32.9)	<0.00001
	Male	376 (51.0)	233 (44.5)	143 (67.1)	
ECOG	≤ 1	603 (81.8)	429 (81.9)	174 (81.7)	0.954
	≥ 2	134 (18.2)	95 (18.1)	39 (18.3)	
eGFR, ml/min/1.73m^2 (mean, SD)	51.8 (27.8)	737 (100.0)	548.7 (29.0)	59.3 (22.6)	<0.00001
History of bladder cancer	No	527 (71.5)	405 (77.3)	122 (57.3)	<0.00001
	Yes	210 (28.5)	119 (22.7)	91 (42.7)	
Tumor location	Renal pelvis	308 (41.8)	232 (44.3)	76 (35.7)	0.014
	Ureter	309 (41.9)	202 (38.5)	107 (50.2)	
	Both	120 (16.3)	90 (17.2)	30 (14.1)	
Hydronephrosis	No	307 (41.7)	187 (35.7)	120 (56.3)	<0.00001
	Yes	430 (58.3)	337 (64.3)	93 (43.7)	
Type of surgery	Nephroureterectomy	630 (85.5)	513 (97.9)	117 (54.9)	<0.00001
	Distal ureterectomy	107 (14.5)	11 (2.1)	96 (45.1)	
Approach of surgery	Open	389 (52.8)	272 (51.9)	117 (54.9)	0.456
	Laparoscopy	348 (47.2)	252 (48.1)	96 (45.1)	
Focality	Unifocal	521 (70.7)	395 (75.4)	126 (59.2)	0.00001
	Multifocal	216 (29.3)	129 (24.6)	87 (40.8)	
Grade	Low	129 (17.5)	93 (17.7)	36 (16.9)	0.784
	High	608 (82.5)	431 (82.3)	177 (83.1)	
pT stage	pTa/Tis	165 (22.4)	77 (14.7)	88 (41.3)	<0.00001
	pT1	150 (20.4)	120 (22.9)	30 (14.1)	
	pT2	154 (20.9)	135 (25.8)	19 (8.9)	
	pT3	225 (30.5)	156 (29.8)	69 (32.4)	
	pT4	43 (5.8)	36 (6.9)	7 (3.3)	
Lymphovascular invasion	No	559 (75.8)	395 (75.4)	164 (77.0)	0.643
	Yes	178 (24.2)	129 (24.6)	49 (23.0)	
pN stage	pN0	277 (37.6)	193 (36.8)	84 (39.4)	0.755
	pNx	393 (53.3)	284 (54.2)	109 (51.2)	
	pN+	67 (9.1)	47 (9.0)	20 (9.4)	
Progression	No	550 (74.6)	388 (74.0)	162 (76.1)	0.570
	Yes	187 (25.4)	136 (26.0)	51 (23.9)	
Death of UTUC	No	607 (82.4)	434 (82.8)	173 (81.2)	0.605
	Yes	130 (17.6)	90 (17.2)	40 (18.8)	
All-cause death	No	537 (72.9)	379 (72.3)	158 (74.2)	0.609
	Yes	200 (27.1)	145 (27.7)	55 (25.8)	

Table S2. Univariate and multivariate analyses of progression-free, cancer-specific and overall survival in 737 patients with UTUC (using sodium level as a continuous variable).

Variable	Progression-free Survival						Cancer-specific Survival						Overall Survival					
	Univariate analysis			Multivariate analysis			Univariate analysis			Multivariate analysis			Univariate analysis			Multivariate analysis		
	HR	95% CI	*p* value	HR	95% CI	*p* value	HR	95% CI	*p* value	HR	95% CI	*p* value	HR	95% CI	*p* value	HR	95% CI	*p* value
Age (continuous)	1.016	1.002-1.031	0.026	1.007	0.992-1.023	0.349	1.029	1.011-1.048	0.001	1.018	0.999-1.037	0.063	1.030	1.016-1.045	0.00005	1.019	1.004-1.034	0.011
Gender																		
Female	1		0.096	1		0.714	1		0.086	1		0.839	1		0.051	1		0.589
Male	1.277	0.957-1.704		1.058	0.782-1.431		1.355	0.958-1.916		1.039	0.721-1.496		1.320	0.999-1.745		1.084	0.810-1.450	
Region																		
Taiwan	1		0.385	1		0.277	1		0.080	1		0.054	1		0.222	1		0.199
U.S.	1.154	0.836-1.593		1.261	0.830-1.917		1.395	0.960-2.026		1.620	0.992-2.648		1.214	0.889-1.658		1.309	0.868-1.974	
ECOG																		
≤ 1	1		0.029	1		0.048	1		0.035	1		0.372	1		<0.00001	1		0.001
≥ 2	1.464	1.041-2.059		1.441	1.003-2.070		1.545	1.031-2.316		1.224	0.785-1.908		2.129	1.568-2.890		1.791	1.287-2.494	
eGFR (continuous)	0.998	0.993-1.004	0.534	0.999	0.993-1.006	0.864	0.997	0.991-1.003	0.353	0.999	0.991-1.007	0.829	0.997	0.992-1.002	0.269	1.000	0.994-1.005	0.874
History of bladder cancer																		
No	1		0.247	1		0.149	1		0.195	1		0.143	1		0.270	1		0.259
Yes	1.201	0.881-1.639		1.276	0.917-1.776		1.276	0.882-1.844		1.344	0.904-1.997		1.186	0.875-1.607		1.206	0.871-1.670	
Tumor location																		
Renal pelvis	1		0.001	1		0.152	1		0.00004	1		0.148	1		<0.00001	1		0.243
Ureter	1.106	0.796-1.538		1.194	0.814-1.752		1.069	0.710-1.610		1.029	0.640-1.654		1.193	0.861-1.653		1.136	0.781-1.654	
Both	1.970	1.351-2.872		1.653	0.995-2.747		2.469	1.600-3.812		1.735	0.951-3.165		2.352	1.641-3.371		1.525	0.932-2.496	
Hydronephrosis																		
No	1		0.015	1		0.753	1		0.035	1		0.106	1		0.033	1		0.175
Yes	1.457	1.076-1.973		1.057	0.748-1.493		1.463	1.027-2.084		1.433	0.927-2.217		1.361	1.025-1.806		1.268	0.900-1.786	
Type of surgery																		
Nephroureterectomy	1		0.110	1		0.531	1		0.248	1		0.761	1		0.132	1		0.551
Distal ureterectomy	0.666	0.404-1.097		0.816	0.432-1.542		0.705	0.389-1.277		0.887	0.409-1.924		0.689	0.424-1.118		0.828	0.445-1.540	
Approach of surgery																		
Open	1		0.023	1		0.503	1		0.004	1		0.758	1		0.008	1		0.457
Laparoscopy	0.712	0.531-0.955		1.129	0.792-1.609		0.586	0.408-0.841		1.074	0.682-1.692		0.679	0.510-0.904		1.141	0.806-1.617	
Focality																		
Unifocal	1		0.0004	1		0.812	1		0.0001	1		0.995	1		0.00003	1		0.600
Multifocal	1.712	1.273-2.303		1.050	0.703-1.569		1.994	1.407-2.827		1.001	0.608-1.650		1.830	1.378-2.430		1.114	0.744-1.669	
Grade																		
Low	1		<0.00001	1		0.027	1		0.00003	1		0.196	1		<0.00001	1		0.120
High	5.517	2.822-10.786		2.230	1.096-4.536		5.844	2.573-13.274		1.788	0.741-4.314		3.186	1.934-5.247		1.542	0.893-2.664	
pT stage																		
pTa/Tis	1		<0.00001	1		<0.00001	1		<0.00001	1		<0.00001	1		<0.00001	1		<0.00001
pT1	1.730	0.823-3.637		1.683	0.784-3.617		0.938	0.329-2.675		0.939	0.322-2.744		0.921	0.495-1.713		0.969	0.510-1.840	
pT2	3.179	1.614-6.260		2.538	1.236-5.211		2.455	1.025-5.879		2.066	0.819-5.215		1.705	0.987-2.946		1.507	0.834-2.724	
pT3	7.450	3.988-13.916		4.750	2.420-9.326		8.600	3.959-18.681		5.475	2.352-12.740		3.961	2.446-6.413		2.915	1.693-5.018	
pT4	18.286	9.086-36.802		6.257	2.717-14.409		26.655	11.503-61.764		8.215	2.966-22.754		10.728	6.025-19.102		4.098	1.952-8.602	
Lymphovascular invasion																		
No	1		<0.00001	1		0.014	1		<0.00001	1		0.014	1		<0.00001	1		0.082
Yes	3.253	2.437-4.343		1.531	1.091-2.149		4.055	2.867-5.735		1.653	1.106-2.472		2.646	1.987-3.524		1.350	0.963-1.892	
pN stage																		
pN0	1		<0.00001	1		0.003	1		0.00001	1		0.067	1		<0.00001	1		0.041
pNx	0.805	0.581-1.116		0.912	0.652-1.275		0.804	0.539-1.198		0.896	0.594-1.352		0.790	0.580-1.076		0.897	0.652-1.234	
pN+	4.287	2.892-6.354		1.951	1.221-3.119		4.818	3.038-7.641		1.665	0.955-2.902		3.441	2.307-5.134		1.619	1.004-2.610	
Sodium (continuous)	0.950	0.919-0.982	0.002	0.964	0.929-1.000	0.049	0.944	0.906-0.982	0.005	0.953	0.910-0.997	0.037	0.945	0.915-0.977	0.001	0.966	0.932-1.002	0.066

HR hazard ratio, *CI* confidence interval, *ECOG* Eastern Cooperative Oncology Group, *eGFR* estimated glomerular filtration rate
